# RNA editing contributes to epitranscriptome diversity in chronic lymphocytic leukemia

**DOI:** 10.1038/s41375-020-0995-6

**Published:** 2020-07-30

**Authors:** Franz J. Gassner, Nadja Zaborsky, Ilana Buchumenski, Erez Y. Levanon, Matthias Gatterbauer, Maria Schubert, Stefanie Rauscher, Daniel Hebenstreit, Ferran Nadeu, Elias Campo, Alexander Egle, Richard Greil, Roland Geisberger

**Affiliations:** 1 Department of Internal Medicine III with Haematology, Medical Oncology, Haemostaseology, Infectiology and Rheumatology, Oncologic Center, Salzburg Cancer Research Institute—Laboratory for Immunological and Molecular Cancer Research (SCRI-LIMCR), Paracelsus Medical University, Salzburg, Austria; 2 Cancer Cluster Salzburg, Salzburg, Austria; 3 The Mina and Everard Goodman Faculty of Life Sciences, Bar-Ilan University, Ramat-Gan, 5290002 Israel; 4 Department of Biosciences, Paris Lodron University of Salzburg, Salzburg, Austria; 5 School of Life Sciences, University of Warwick, Coventry, UK; 6 Molecular Pathology of Lymphoid Neoplasms Program, Institut d’Investigacions Biomèdiques August Pi i Sunyer (IDIBAPS), Barcelona, Spain; 7 Centro de Investigación Biomédica en Red de Cáncer (CIBERONC), Madrid, Spain; 8 Hematopathology Section, Hospital Clínic of Barcelona, University of Barcelona, Barcelona, Spain; 9 Salzburg Cancer Research Institute—Center for Clinical Cancer and Immunology Trials (CCCIT), Salzburg, Austria; 10 Austrian Group for Medical Tumor Therapy (AGMT), Salzburg, Austria

**Keywords:** Cancer genetics, Cancer genetics

## Abstract

RNA editing—primarily conversion of adenosine to inosine (A > I)—is a widespread posttranscriptional mechanism, mediated by Adenosine Deaminases acting on RNA (ADAR) enzymes to alter the RNA sequence of primary transcripts. Hence, in addition to somatic mutations and alternative RNA splicing, RNA editing can be a further source for recoding events. Although RNA editing has been detected in many solid cancers and normal tissue, RNA editing in chronic lymphocytic leukemia (CLL) has not been addressed so far. We determined global RNA editing and recurrent, recoding RNA editing events from matched RNA-sequencing and whole exome sequencing data in CLL samples from 45 untreated patients. RNA editing was verified in a validation cohort of 98 CLL patients and revealed substantially altered RNA editing profiles in CLL compared with normal B cells. We further found that RNA editing patterns were prognostically relevant. Finally, we showed that ADAR knockout decreased steady state viability of MEC1 cells and made them more susceptible to treatment with fludarabine and ibrutinib in vitro. We propose that RNA editing contributes to the pathophysiology of CLL and targeting the RNA editing machinery could be a future strategy to maximize treatment efficacy.

## Introduction

Recent data from whole-exome and whole-genome sequencing approaches revealed a complex genomic landscape for many cancer entities [1–3]. In addition to somatic mutations and alternative splicing, genetic information can also be altered by RNA editing. Generally, RNA editing is the posttranscriptional modification of RNA bases, with adenosine to inosine (A > I) being the most prevailing type of editing [4, 5]. As I is structurally a guanosine (G) analog, A to I editing can cause amino acid changes, retargeting of miRNAs and altered RNA splicing patterns. A to I editing occurs in almost any tissue from metazoan organisms and is catalyzed by adenosine deaminases that act on RNA (ADARs), which, in mammals, comprise ADAR1 (or ADAR), ADAR2 (or ADARB1) and catalytically-inactive ADAR3 family members [6]. ADAR1 has two isoforms, the full length isoform p150, which is driven from an inflammation dependent promoter and the constitutively expressed, N-terminally truncated isoform p110. Increased RNA editing was recently detected in many solid cancer entities compared with healthy tissue, which has been attributed to an increased expression of ADAR1 in cancer, particularly the inflammation dependent isoform p150 [7–9]. Global RNA editing measurements from hundreds of cancer samples revealed millions of editing sites, most of them occurring in transcribed noncoding *Alu*-sites. *Alu* elements are short, repetitive DNA stretches with more than one million copies distributed throughout the genome. Pairs of inverted copies of *Alu* elements are preferred targets for ADARs [10]. Consequently, editing of *Alu* elements has been used as an indicator for global editing activity [7, 8]. However, editing in coding regions has also been detected and some recurrent editing sites affecting coding regions were reported to influence cancer development and progression [11–13].

Chronic lymphocytic leukemia (CLL) is a highly heterogeneous disease [14] and, accordingly, recent high throughput sequencing studies identified a large panel of around 80 putative driver genes from more than 1,000 patients, revealing high interpatient mutational heterogeneity [15–17]. However, RNA editing has not yet been investigated in this cancer entity and it is unknown how the complex joint effects from genome mutations/aberrations and RNA editing interrelate with clinical parameters, disease progression and response to therapies. In this study, we analyzed RNA editing from matched exomes and transcriptomes of 45 previously untreated CLL patients. From these data, we established a catalog of recurrent and recoding editing sites in CLL and validated these sites in a second CLL cohort and in normal B cell subsets. Our analysis provides for the first time a detailed insight into RNA editing in CLL and normal B cells, revealing recurrent recoding of conserved sites and its association with clinical parameters in CLL.

## Materials and methods

### Patients and cell lines

Peripheral blood from chemo-naïve CLL patients participating in a previously reported clinical trial (AGMT-REVLIRIT trial, ClinicalTrials.gov Identifier: NCT00738829 and NCT01703364) [18] with first line treatment with lenalidomide in combination with fludarabine and rituximab was collected upon informed consent and ethical approval by the Ethics Committee of the Province of Salzburg (415-E/1287/4–2011, 415-E/1287/8–2011). Sampling was performed prior treatment start and CLL cells were obtained by density gradient centrifugation and B-CLL Cell Isolation kit (Miltenyi Biotec). Cell purity was >90% in all samples. The determination of prognostic markers was performed routinely at our department as described previously [19]. DNA and RNA was purified using DNeasy Blood and Tissue kit or RNeasy Mini kit (both Qiagen), respectively. Patient details and type of analysis are given in supporting Table 1. Primers are listed in supporting Table S2. RNA/DNA sequencing data from AGMT-REVLIRIT patients and MEC1 cells are available under BioProject PRJNA540189 at https://www.ncbi.nlm.nih.gov/sra. In addition, RNA sequencing and clinical data from CLL samples and normal B cells were downloaded from the European Genome-phenome Archive (EGAS00001000374) [20].

## Results

### Characteristics of patients

We studied RNA editing in 45 previously untreated CLL patients prior to first line therapy with fludarabine/rituximab combined with escalating doses of lenalidomide (AGMT-REVLIRIT study [18], see Material and Methods). The mean age of patients was 66 years (range 43–80). 20 of 45 patients (44%) were female and 25 were male (56%). 19 patients had a mutated IGHV (42%), 21 were IGHV unmutated (47%) and five patients (11%) had undefined IGHV status. 13 patients (29%) had ‘high risk’ FISH cytogenetics with 17p and/or 11q deletions (Table 1 and supporting Table S1). We were able to isolate matched RNA and genomic DNA from all 45 patients.

**Table 1:** Patient characteristics of CLL cohorts.

Parameters	AGMT-REVLIRIT cohort [18]	CLL validation cohort [20]
Total number (%)	45 (100)	98 (100)
Sex		
Male (%)	25 (56)	68 (69)
Female (%)	20 (44)	30 (31)
Age (years)		
Mean	65.8	66.7
Range	43–80	38–89
Duration of disease (years)		
Mean	3.8	5.4
Range	0–10.3	0–21.9
RAI stage at diagnosis		
nda	2 (4)	
I	7 (16)	
II	16 (36)	
III	12 (27)	
IV	8 (18)	
Binet stage at diagnosis		
nda		2 (2)
A		91 (93)
B		4 (4)
C		1 (1)
Molecular risk parameters		
Unmutated Ig VH	21 (47)	43 (44)
IGHV nda	5 (11)	2 (2)
FISH karyotype		
del11q	9 (20)	14 (14.3)
del13q	29 (64)	51 (52.0)
del17p	4 (9)	4 (4)
trisomy 12	6 (13)	14 (14)
normal karyotype	5 (11)	23 (23)
karyotype nda	1 (2)	2 (2)
Treatment status		
Untreated at sampling	45 (100)	98 (100)
Untreated at last follow up	0 (0)	39 (40)

*IGHV* immunoglobulin variable heavy chain, *FISH* fluorescence in situ hybridization, nda no data available.

### Editing of *Alu* sites correlates with ADAR expression

**Fig. 1:**
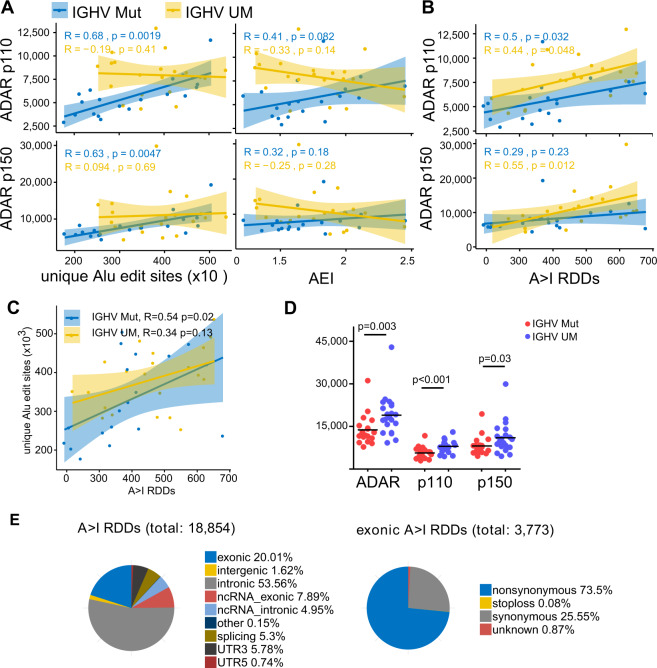
A > I editing in CLL. **a** Unique *Alu* editing sites and *Alu* editing index (AEI) were determined in CLL samples and correlated with normalized expression of ADAR1 (ADAR) isoforms p110 (upper panel) and p150 (lower panel) according to IGHV mutation status. **b** A to I (RNA-DNA single nucleotide differences) RDDs were correlated with ADAR isoforms p110 and p150. **c** A > I RDDs were correlated with unique *Alu* editing sites according to IGHV mutation status. **d** ADAR mRNA levels as well as ADAR isoforms p110 and p150 levels were determined for IGHV mutated and unmutated CLL samples. Significances calculated using unpaired *t* test. **e** Functional consequences of A > I RDDs are depicted as pie charts for total A > I editing events and for exonic A > I RDDs.

First, we calculated RNA editing within *Alu* regions (repetitive, mostly noncoding elements in the genome) in CLL samples from RNA sequencing data as previously described [7]. As 99% of global RNA editing is confined to *Alu* regions, RNA editing in *Alu* sites reflects the overall A > I RNA editing activity of a particular sample. We measured all edited adenosine within *Alu* elements (unique *Alu* editing sites) as well as the averaged editing level across all *Alu* adenosines, weighted by their relative expression (*Alu* editing index; AEI) [7]. We detected unique editing sites ranging from 177,720 up to 537,995 events per sample and an AEI ranging from 1.22 to 2.45 arbitrary units in our CLL cohort. In line with previous results from other cancer entities, we found that editing directly correlated with ADAR mRNA expression in the IGHV mutated samples, irrespective of the ADAR isoform (p110 or p150) expressed. However, correlation of AEI with p110 was more significant compared with p150 (Fig. 1a). Surprisingly, unique *Alu* editing sites and AEI did not correlate with ADAR p110 or p150 expression in the IGHV unmutated samples (Fig. 1a).

As we observed RNA editing activity to correlate with ADAR levels only in IGHV mutated CLL, we asked whether a set of recently identified editing cofactors [21] would be differentially expressed in IGHV mutated versus unmutated CLL. Indeed, we observed that three editing factors (CCDC9, TIMM17A, and MRPL47) were significantly differentially expressed according to IGHV mutation status (false discovery rate adjusted *p* < 0.05; supporting Table S3).

### RNA editing in coding regions

**Fig. 2:**
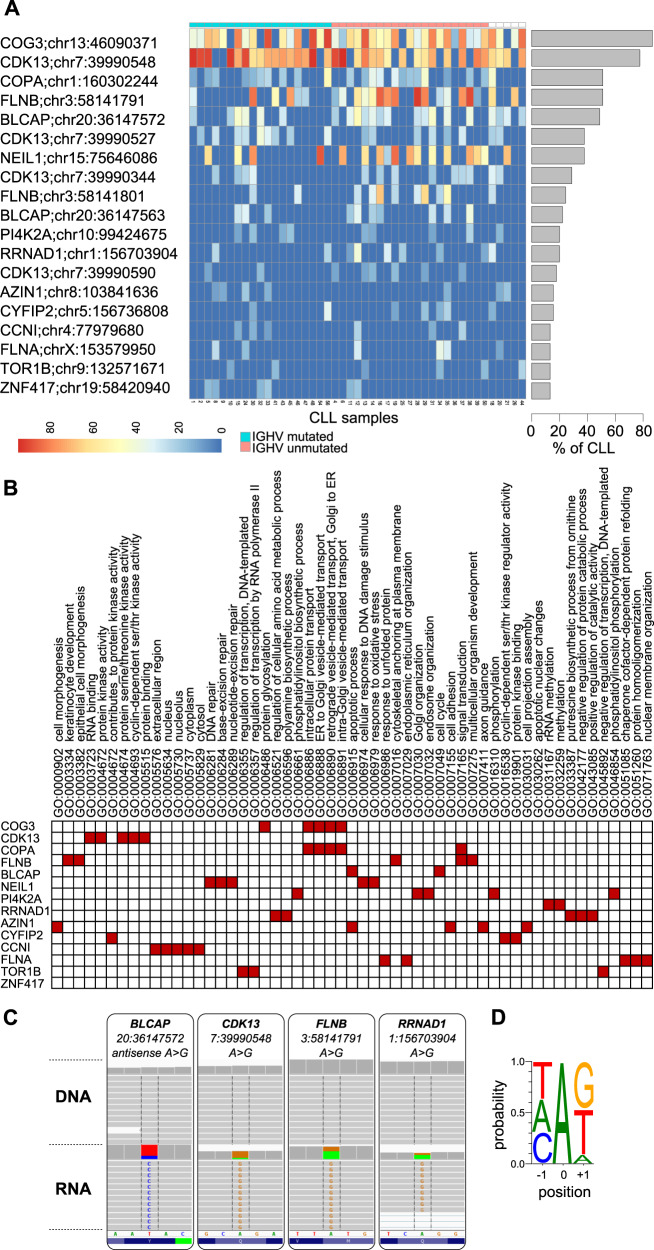
Recurrent recoding A > I editing in CLL. **a** Heat map of editing frequencies of 19 recurrent A > I editing sites within 14 genes in CLL cells from the AGMT-REVLIRIT cohort [18]. Patient IDs are depicted below the heat map. **b** Mapping of 14 edited genes to biological pathways. **c** Integrative Integrative Genomics Viewer screenshot (http://software.broadinstitute.org/software/igv/) of RNA-seq and WES data of exemplary edited genes. **d** Sequence context of A > I editing sites from editing sites shown in (**a**).

RNA editing in *Alu* repeats is a proxy for global RNA editing activity, but gives little information on recoding events, as those are mostly located outside *Alu* elements. In order to determine RNA editing in coding regions, we next screened for RNA-DNA single nucleotide differences (RDDs) from matched RNA-sequencing/whole-exome sequencing (WES) data from our 45 patients. Screening for differences in matched RNA/DNA pairs revealed a total of 80,150 RDDs in 45 samples. Similar to a previous report [22], our RDD analysis yielded all possible base changes in recoding and nonrecoding events (Fig. S1); however, RDDs present in *Alu* regions (6864 events) as determined by the UCSC repeat masker track, were mostly of the A > I type, consistent with the finding that A > I editing occurs predominantly in *Alu* regions (Fig. S1). Among all RDDs, we found between 196 and 686 A > I editing events per sample. The number of A > I editing sites correlated particularly with ADAR p110 expression irrespective of the IGHV mutation status (Fig. 1b) and with the number of unique editing sites in *Alu* regions particularly in IGHV mutated samples (Fig. 1c). Notably, ADAR levels, particularly isoform p110, were generally higher in IGHV unmutated CLL samples (Fig. 1d). From the total of 18,854 A > I RDDs from all 45 CLL, 3773 (20%) mapped to exonic regions of which 2773 (73.5%) resulted in amino acid changes (Fig. 1e). From the 2773 nonsynonymous editing events, we found a set of 19 editing sites confined to 14 genes that were recurrently edited in at least 5 out of 45 patients, with no apparent IGHV specific differences (Fig. 2a, supporting Table S4). These genes are associated with 55 diverse biological pathways (Fig. 2b).The recurrent editing sites were individually checked for ambiguity or misalignment using BLAST search and for artefacts using Integrative Genome Viewer (IGV, Fig. 2c). Except for editing of ZNF417 (chr19:58420940), we found all of these sites already described as A > I editing targets in published datasets [23–25], and they matched the consensus ADAR deamination motif with preference for underrepresentation of G bases at the −1 position and overrepresentation of G at the +1 position [26] (Fig. 2d). Of note, we did not observe apparent correlations of editing frequency with expression levels of the respective genes, except for FLNB, NEIL1, and PI4K2A editing, which showed a weak positive correlation with expression (Fig. S2).

### Recurrent RNA editing patterns are different between CLL and normal B cells

Next, we accessed RNA-seq data from a validation cohort of 98 CLL samples from Ferreira et al. (Table 1) [20], which included normal naïve B cells as well as class switched and nonclass switched memory B cells (*n* = 9 for each normal B cell subset), allowing comparison of CLL RNA editing with that from different normal B cell subsets. From these RNA-seq data, we extracted A/G variant frequencies at the 19 editing sites. While in most samples from the CLL cohort from Ferreira et al the coverage of the CDK13 gene was too low for assessing RNA editing - and we hence excluded the four CDK13 sites from all further analyses—we found that the remaining 15 out of these 19 sites were also robustly edited in the validation cohort. Moreover, depending on the IGHV mutation status, editing depths (i.e., editing frequencies) from most of these sites were significantly different compared with normal B cell subsets, indicating aberrant RNA editing in CLL. While normal B cell subsets had distinctive RNA editing depths for each site, RNA editing in CLL cells was highly variable (Fig. S3A). The global RNA editing activity as measured by the AEI was significantly increased in naïve B cells compared with CLL cells and to other normal B cell subsets (Fig. S3B). In addition, the AEI was significantly lower in IGHV unmutated CLL cases compared with IGHV mutated samples (Fig. S3C) and the variance from CLL cells was significantly greater than that from normal cells (*F* = 4.84, *p* = 0.00003), underpinning increased variability in RNA editing activity in malignant cells. Conversely, expression of ADAR isoform p150 is significantly higher in CLL compared with normal B cells and corroboratively to the AGMT-REVLIRIT cohort, ADAR expression is slightly increased in IGHV unmutated samples (Fig. S3D). Furthermore, in line with the AGMT-REVLIRIT cohort (Fig. S2), we again observed no apparent correlation of editing depths with expression levels, neither for CLL cells (Fig. S4) nor for normal B cells (Fig. S5).

### Clinical relevance of A > I RNA editing

**Fig. 3:**
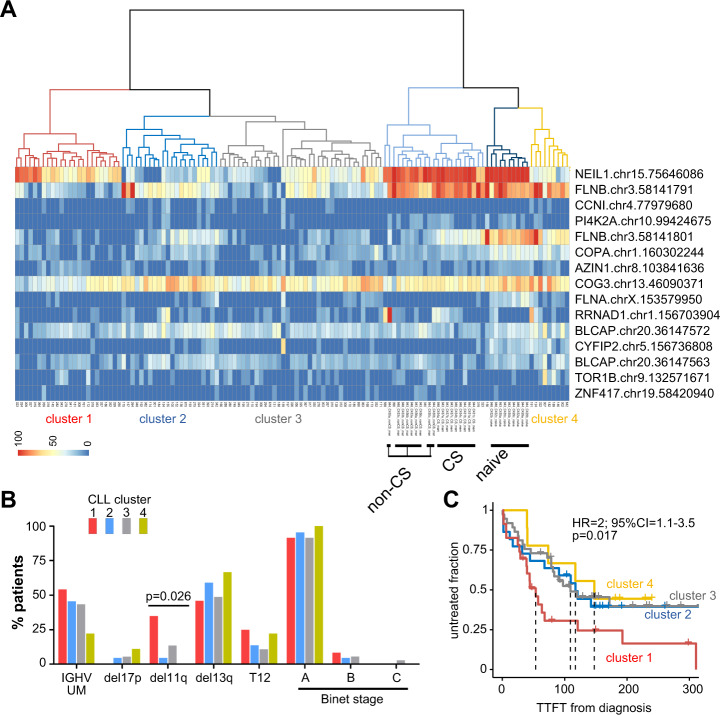
RNA editing in CLL datasets. **a** Hierarchical clustering of recurrently edited sites from CLL and normal B cell subsets (non-CS, CS and naïve B cells) from the Ferreira cohort [20]. **b** Distribution of the clinical features in the four CLL clusters defined by RNA editing in (**a**). **c** Time to first treatment of patients assigned to the four RNA editing clusters. Univariate analysis for cluster 1 versus noncluster 1 patients is indicated in graph. (non-CS non-class switched memory B cells, CS class switched memory B cells, TTFT time to first treatment, HR hazard ratio, CI confidence interval, p: significance).

Next, we performed hierarchical clustering of editing sites from the Ferreira cohort [20]. This resulted in a robust separation of normal naive B cells, memory B cell subsets and four groups of CLL samples, which differed in number and editing depths of the recurrent editing events (Fig. 3a). This again confirmed specific RNA editing patterns for each normal B cell subset and aberrant RNA editing in CLL. The four groups defined by clustering of RNA editing patterns were largely independent from IGHV mutation status, Binet staging and chromosomal aberrations, except for cluster 1 showing association with increased presence of del11q (Fig. 3b). In this cluster 1, the AEI was significantly decreased compared with other RNA editing clusters and compared with normal naive B cell subsets (Fig. S6A). However, ADAR isoform expression was only slightly different between the individual clusters (Fig. S6B). Moreover, patients within cluster 1 had significantly shortened time to first treatment (TTFT) compared with other patients. For time from diagnosis, the median TTFT for cluster 1 was 53.6 versus 117.3 months for noncluster 1 patients, HR = 2, 95%CI = 1.1–3.5, *p* = 0.017 (log-rank test; Fig. 3c). However, in multivariate analysis, IGHV mutation status remained the most powerful independent prognostic parameter in this cohort (supporting Table S5).

To validate the results from the Ferreira cohort, we performed hierarchical clustering of editing sites from the AGMT-REVLIRIT cohort (*n* = 45) [18], which yielded 7 distinct RNA editing clusters (Fig. S7A). In this analysis, cluster 5 is highly similar to cluster 1 from the Ferreira cohort, characterized by high NEIL1 editing, intermediate RNA editing of COG3 and low editing of any other site. Again, this cluster showed the shortest time to first treatment of 31.9 months compared with 42 months for all noncluster 5 patients, although this was not statistically significant (HR = 2.03; 95%CI = 0.88–4.7; *p* = 0.09; log rank test, Fig. S7B). However, in multivariate analysis, neither IGHV mutation status, nor cytogenetics remained a significant independent prognostic parameter, probably due to small sample size of this cohort (supporting Table S6).

**Fig. 4:**
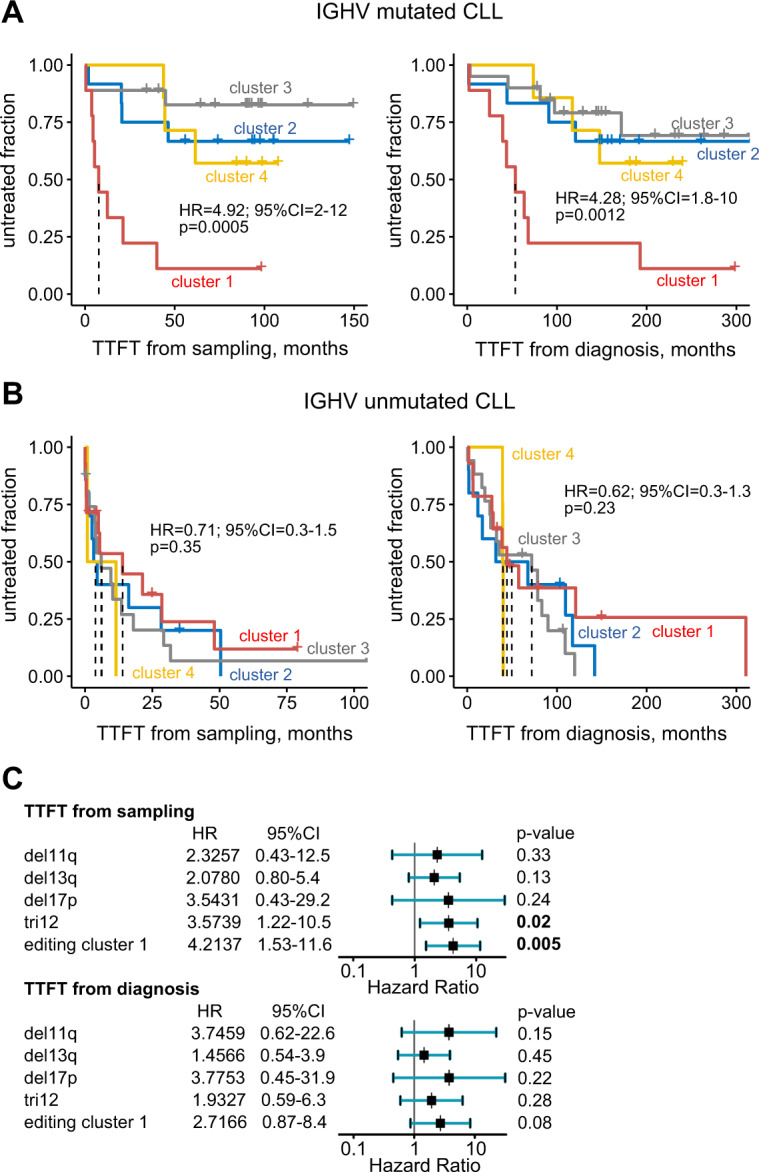
RNA editing and time to first treatment. **a** Time to first treatment is shown for time from sampling (left graph) and time from diagnosis (right graph) for IGHV mutated (**a**) and IGHV unmutated samples (**b**) from the Ferreira cohort [20]. Univariate analyses for cluster 1 versus noncluster 1 patients are indicated in graphs. **c** Multivariate analysis of time to first treatment from sampling and diagnosis of IGHV mutated patients according to the indicated risk parameters. (TTFT time to first treatment, HR Hazard Ratio CI confidence interval, p: significance).

Strikingly, stratifying patients from the Ferreira cohort according to IGHV mutation status revealed that editing-cluster 1 patients had significantly shortened time to first treatment particularly within the IGHV mutated cases (Fig. 4a). As RNA editing is potentially a dynamic process, which may evolve with disease progression, we next calculated treatment-free intervals for patients considering time from diagnosis versus time from sampling. Thereby we found that the shortened treatment-free interval for IGHV mutated patients within editing cluster 1 was even more significant for time from sampling compared with time from diagnosis. For time from sampling, the median TTFT for cluster 1 IGHV mutated patients was 7.68 versus not reached for noncluster 1 IGHV mutated patients, HR = 4.92, 95%CI = 2–12, *p* = 0.0005 (log-rank test; Fig. 4a left graph). For time from diagnosis, the median TTFT for cluster 1 IGHV mutated patients was 53.6 versus not reached for noncluster 1 IGHV mutated patients, HR = 4.28, 95%CI = 1.8–10, *p* = 0.0012 (log-rank test; Fig. 4a right graph). For IGHV unmutated patients, RNA editing clusters did not show any prognostic relevance (Fig. 4b). In multivariate analyses, the RNA-editing cluster remained the most significant independent prognostic factor amongst chromosomal aberrations del11q, del17p, del13q, and trisomy12 for IGHV mutated patients (Fig. 4c).

In the AGMT-REVLIRIT cohort, only two patients from RNA editing cluster five were IGHV mutated, rendering the log-rank test not significant (Fig. S8). As for patients from the AGMT-REVLIRIT cohort sampling was done at treatment start, the treatment-free interval from sampling date was not calculable. Notably, we also analyzed time to progression upon treatment of patients in the two CLL cohorts. While the RNA editing cluster did not significantly predict progression free survival (PFS) in the Ferreira cohort, PFS was prolonged in patients with RNA editing cluster 5 from the AGMT-REVLIRIT cohort (Fig. S9).

To identify possible reasons for the differential editing activity in normal versus malignant cells, we again analyzed putative cofactors involved in editing [21]. We found many editing factors differentially expressed between normal B and CLL cells at high significance. In addition, six factors were differentially expressed in CLL according to IGHV mutation status. However, we only found a few factors differentially expressed between individual editing clusters (supporting Table S7). In addition, some RNA binding proteins, which were recently shown to affect RNA editing [27] were found to be differentially expressed within the respective CLL clusters and according to IGHV status of CLL (supporting Table S8).

### ADAR deficiency sensitizes toward CLL treatment in vitro

**Fig. 5:**
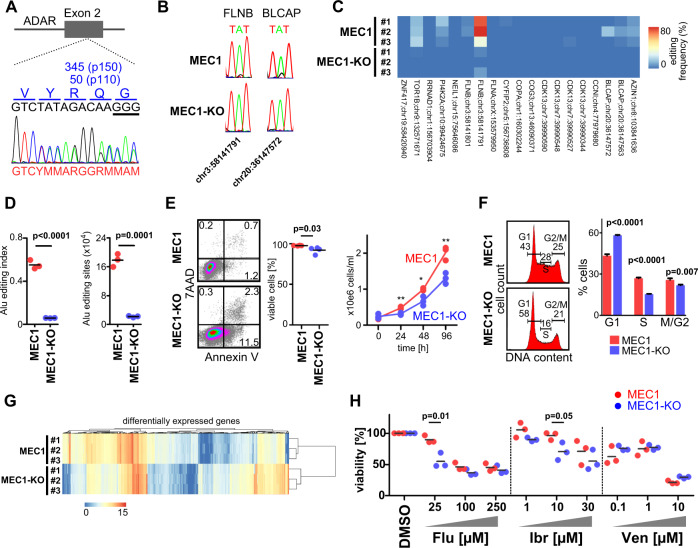
ADAR knockout in MEC1 cells sensitizes toward in vitro treatment. **a** Schematic representation of ADAR exon 2 and DNA/protein sequence of the CRISPR/Cas9 target site (protospacer adjacent motif is underlined) for the two ADAR isoforms p110 and p150. Sanger sequence of the target site from MEC1 ADAR-knockout cells is shown below (Y = C or T; M = A or C; R = G or A). **b** A > I editing of FLNB and BLCAP in MEC1 and MEC1 ADAR knockout (MEC1-KO) cells. **c** Heat map of editing frequencies of 19 recurrent A > I editing sites in MEC1 and MEC1 ADAR knockout cells. **d** Unique *Alu* editing sites and *Alu* editing index (AEI) for MEC1 and MEC1 ADAR knockout cells. **e** Representative viability stains (measured by flow cytometry and 7AAD/AnnexinV) and dot plot from *n* = 4 independent experiments (left graph) and longitudinal cell counts (right graph, *n* = 3) from MEC1 and MEC1 ADAR knockout cells. **f** Representative cell cycle stains of MEC1 and MEC1 ADAR knockout cells and statistics from *n* = 3 independent experiments (mean ± SD). **g** Heat map of differentially expressed genes in MEC1 versus MEC1 ADAR knockout cells. **h** MEC1 and MEC1 ADAR knockout cells were treated with different doses of indicated drugs in vitro for 72 h followed by viability measurements using XTT assays (Flu: fludarabine; Ibr: ibrutinib; Ven: venetoclax). Viability of controls (DMSO treated cells) were set to 100%. Significances calculated using unpaired *t* test (n values indicate independent experiments; horizontal lines in dot plots show mean values); *p = 0.01; **p < 0.01.

As our data showed that RNA editing patterns correlate with prognosis in CLL, we tested whether RNA editing activity directly affects viability of CLL cells and drug sensitivity. Therefore, we knocked out ADAR in the prolymphocytic CLL cell line MEC1 [28] (Fig. 5a). We verified the knockout by Sanger sequencing (Fig. 5a) and additionally monitored RNA editing of selected editing sites in MEC1 versus MEC1 ADAR-knockout cells by Sanger-sequencing. From six genes analysed (CDK13, CCNI, FLNB, COG3, COPA, BLCAP), we detected robust editing of two sites in MEC1 cells (FLNB, chr3:58141791; BLCAP, chr20:36147572), which remained unedited in ADAR-knockout cells, verifying that these editing events were indeed ADAR dependent (Fig. 5b). To more thoroughly investigate ADAR dependent RNA editing in MEC1, we performed RNA-seq of MEC1 and MEC1 ADAR-knockout cells. Thereby, we found that from our set of 19 recurrent editing sites, AZIN1, PI4K2A and TOR1B sites were also edited in MEC1 cells but not in the knockout cells (Fig. 5c). Furthermore, the AEI and unique *Alu* editing sites were dramatically decreased in absence of ADAR (Fig. 5d). In addition, we screened MEC1 cells for editing at sites recently described in a glioblastoma cell line and primary breast cancer samples [29], which revealed that many of these sites were also edited in MEC1 cells in an ADAR dependent manner (supporting Table 9).

We further noticed that knockout of ADAR in MEC1 cells already resulted in a slight and significant decrease of viability, proliferation, and cells in S/M/G2 phase of the cell cycle (Fig. 5e, f). Furthermore, our RNA-seq data revealed many differentially expressed genes between wildtype and knockout cells (Fig. 5g, supporting Table 10) resulting in significantly over- and underrepresented gene sets and pathways in gene ontology and KEGG (Kyoto Encyclopedia of Genes and Genomes) pathway analysis. However, we did not find significant differences in cell cycle, apoptosis, or IFN signaling pathways (supporting Tables S11 and S12). Notably, RNA-seq revealed that both ADAR isoforms p110 and p150 were expressed at similar levels in MEC1 cells (Fig. S10A), which roughly resembles p110/p150 ratios found in primary CLL samples (Figs. 1d and  S10B). Finally, we tested whether ADAR knockout cells showed different sensitivity toward current treatments in vitro. Therefore, we incubated wildtype and knockout MEC1 cells with different doses of fludarabine, and with the BTK inhibitor ibrutinib and the BCL2 inhibitor venetoclax and monitored viability of cells. We found that knockout cells showed increased sensitivity toward treatment particularly with fludarabine and ibrutinib (Fig. 5h). While MEC1 cells were resistant to 25 µM fludarabine and 10 µM ibrutinib, the ADAR knockout cells showed significantly decreased viability at these concentrations.

## Discussion

A to I RNA editing was recently shown to substantially contribute to transcript diversification in health and disease and an increased global editing activity (determined by *Alu*-editing) was shown to frequently associate with poor clinical outcome in solid cancer [7]. In contrast to somatic mutations and genome aberrations, RNA editing enables alterations of genome information in a very dynamic and flexible way, as editing depths may vary between daughter cells and in dependence of interaction with microenvironmental niches. In this study, we describe for the first time RNA editing in CLL and defined 19 recurrent, nonsynonymous editing sites within 14 genes. Although editing sites were shared between normal and malignant cells, the editing pattern was highly characteristic for CLL or normal subsets, revealing that while editing is homogenous in normal B cell subsets, it became very heterogeneous with high variance in malignant cells. As RNA editing is not simply determined by ADAR activity but also by many RNA binding proteins, we assume that subtle changes in their expression patterns likely contribute to the observed aberrant RNA editing activity in CLL [27]. This assumption is supported by our finding that many described editing cofactors [21, 27] were differentially expressed between CLL subgroups and also compared with normal B cells. In addition, ADAR isoform expression is different between CLL and B cells, with CLL samples showing higher ADAR p150 levels than B cells. This could be likely due to a more inflammatory microenvironment in CLL patients, which could also contribute to the aberrant RNA editing patterns observed in CLL. Surprisingly, ADAR p110/p150 ratios were slightly different between the two CLL cohorts, which could be due to differences in RNA preparation, handling, library preparation or sequencing.

RNA editing patterns were highly specific for normal naïve and memory B cells, corroborating recent results that ADAR mediated RNA editing is required for normal B cell development [30]. For CLL cells, we defined a specific RNA editing cluster, which was associated with shortened time to first treatment in two CLL cohorts, particularly in IGHV mutated cases. Strikingly, the global editing activity as determined by the AEI was lower in patients with shortened TTFT and lower compared with normal naive B cells. This is in contrast to many solid cancers, where RNA editing rates were higher in malignant versus healthy tissue [7]. Increased RNA editing in cancer is thought to dampen anti-cancer immunity, as editing of *Alu*-elements prevents their binding to dsRNA sensors, which leads to a robust interferon response [5, 31]. In line with this, antitumor immunity was fundamentally increased in mouse models using ADAR-deficient tumor transplants [32, 33]. In this regard, the overall low RNA editing activity in CLL could reflect a general low anti-cancer immunity and low immunologic pressure in CLL, which would be in line with the observation that CLL patients generally do not respond to immune reactivation using immune checkpoint inhibitors [34]. Hence, it would be interesting to test whether ADAR inhibition in CLL would somehow improve anti-CLL immune responses in patients.

So far, many cancer cell lines have shown increased vulnerability toward type-I interferon signaling upon ablation of ADAR [35]. Results from the present study showed that ADAR loss also increases sensitivity toward fludarabine and ibrutinib in vitro, revealing that therapeutic inhibition of ADAR not only improves antitumor immunity but likely also potentiates drug efficacy. The observed synergism between ADAR ablation and drug treatment may either be based on potentiation of the IFN-response, which is elicited upon ADAR loss as well as upon many treatments [36], or alternatively, recoding of edited genes may affect distinct biological pathways, synergizing with particular drug effects. Surprisingly, although we found many differentially expressed genes and gene sets upon ADAR loss in MEC1 cells, our data did not show particular changes in IFN genes and IFN pathways. This might indicate that gene expression differences and decreased viability upon ADAR loss would rather result from non-synonymously edited transcripts than from an increased IFN-response upon absence of *Alu* editing in MEC1 cells.

Summarizing, we showed that RNA editing substantially contributes to protein recoding in CLL. Our data on MEC1 cells further suggest that interference with ADAR function renders CLL cells more susceptible to distinct therapeutic regimens in vitro, which makes ADAR an interesting target for future combination-treatment strategies.

## Supplementary information

supplemental methods

supplemental figure and table legends

Figure S1

Figure S2

Figure S3

Figure S4

Figure S5

Figure S6

Figure S7

Figure S8

Figure S9

Figure S10

Table S1

Table S2

Table S3

Table S4

Table S5

Table S6

Table S7

Table S8

Table S9

Table S10

Table S11

Table S12
