# Supplementary material for: RNA editing contributes to epitranscriptome diversity in chronic lymphocytic leukemia
Source: Leukemia. 2020 Jul 30;35(4):1053–63. doi: 10.1038/s41375-020-0995-6 (PMC8024191; doi:10.1038/s41375-020-0995-6)
Supplement: Supplementary file 1 — supplemental methods [file 41375_2020_995_MOESM1_ESM.docx]

Supplemental methods

Cell lines

MEC1 cells[1] were cultured in RPMI medium supplemented with 10% FCS (Gibco). MEC1 cells were authenticated by DNA fingerprinting and cytometrical analysis of surface markers (DSMZ). After purchase, stocks of MEC1 cells were frozen and passaged less than 6 months. An ADAR knockout in MEC1 cells was generated by transfecting cells (Amaxa nucleofection, Lonza) with a pGuide-it-ZsGreen1 Vector (Clontech) based construct encoding sgRNA specific for ADAR (5’-AGGGGGATGTCTATAGACAA-3’). 3 days after transfection, GFP positive cells were single cell sorted into 96 well plates (FACS AriaIII, Beckton Dickinson) and expanded. Individual knockout clones were analysed by PCR using primer RG1435 (5’-TCTTCTTGAGCCTTTTATTGCAGTC-3’) and RG1436 (5’-CCATTGTAATGAACAGGTGGTTTCA-3’) and Sanger sequencing using primer RG1435.

Viability of cells was determined by flow cytometry (Gallios, Beckman Coulter) using Annexin-V-FITC (invitrogen)/7-AAD (eBioscience) staining and using XTT assays (Cell Signaling) upon addition of fludarabine, ibrutinib and venetoclax (all Selleckchem) according to the instructions of the manufacturer. Cell counts were determined using an automated cell counter (EVE, NanoEnTek). Cell cycle analysis by DNA content staining of MEC1 and MEC1 ADAR knockout cells was conducted by flow cytometry (Gallios, Beckman Coulter) using Vybrant DyeCycle VioletStain (ThermoFisher) 24h post seeding cells at a density of 0.5x10^6^ cells/ml.

For RNA-seq, total RNA of three batches of separately grown MEC1 and MEC1 ADAR knockout cells was purified using High Pure RNA Isolation kit (Roche). The quality and quantity of isolated total RNA was assessed using the Agilent 4200 TapeStation system (Agilent Technologies) and the Qubit RNA HS Assay Kit (Invitrogen). All samples had a RNA integrity number (RIN) of more than 9.0 showing high quality RNA. 150 ng of each total RNA sample was subjected to mRNA enrichment using the NEBNext poly(A) mRNA Magnetic Isolation Module prior mRNA library preparation using the NEBNext® Ultra™ Directional RNA library Prep Kit for Illumina® (both NEB). Prior sequencing on the in-house NextSeq platform (Illumina), all libraries were qualified and quantified using the Agilent 4200 TapeStation system (Agilent Technologies) and the Qubit dsDNA HS Assay Kits (Invitrogen).

Whole-exome sequencing (WES) of CLL samples

DNA fragmentation, library preparation and WES was performed at GATC Biotech (Germany). Illumina Exome (Agilent SureSelectXT Human All Exon V5) sequencing reads in fastq format were mapped to the human reference genome (hg19) using bowtie2 [2] (v2.1.0, options: –X 10000).

Transcriptome RNA-seq of CLL samples

RNA fragmentation, library preparation and sequencing on Illumina HiSeq 2000/2500 paired end reads were performed at eurofins Genomics (Germany). Transcriptome (Illumina shotgun protocol) sequencing reads in fastq format were trimmed with trimmomatic[3] (v0.33, default settings) and aligned to the hg19 human reference genome using the splice-aware aligner tophat2[4]. A read count matrix was created using “featureCounts”[5] and differential expression analysis was performed using the “edgeR”[6] R package as described in the documentation.

Transcription depth of the p110 and p150 ADAR isoforms was determined by extracting the mean per base coverage in exon 1 of p110 (hg19: chr1:154578483-154578776 and chr1:154600332-154600456) and exon 1 of p150 (chr1:154580469-154580724) of each sample using GATK v4 DepthOfCoverage (www.gatk.broadinstitute.org) from aligned bam-files and the p110/p150 coverage ratio was calculated.

The absolute isoform read counts were calculated for p110 (total ADAR counts * ratio / (ratio + 1)) and p150 (total ADAR counts / (ratio + 1)). The sum of absolute p110 and p150 read counts equals the total ADAR counts for each sample.

Calling RNA-DNA single nucleotide differences (RDDs)

Duplicates in matched exome and transcriptome bam files were marked by picard-tools (v2.2.2, <https://broadinstitute.github.io/picard/>) and the processed bam files were summarized using samtools-0.1.19[7] mpileup. Loci with a mean base quality phred score below 20 or with a coverage of less than 6 reads in either DNA or RNA were omitted. We then used a custom perl script to remove variants that occurred in the first or last base of a read or were strand-biased (present only in either forward or reverse reads). We furthermore omitted variants in deleted or inserted regions and selected only variants uniquely found in RNA by using the somatic variant caller VarScan2 (--min-coverage 8 --min-var-freq 0.05 --strand-filter 1), filtered for high confidence variants (processSomatic, see Koboldt et al[8], “Basic Protocol 2”), and subsequently annotated them using ANNOVAR. A custom perl script and information from the hg19.refseq.bed file retrieved from the UCSC database were used to translate variants in genes with reverse strand orientation. Variants in HLA genes were excluded. All A-to-G variants occurring in at least 5 patients with at least 10% allelic frequency were visually confirmed in IGV and we performed a BLAST search for read sequences harboring A-to-G variants to exclude misalignment errors. Variants with multiple alignments or in repeat regions were omitted. Recurrent editing sites were extracted from RNA-seq data EGAS00001000374. Therefore, fastq files were downloaded from the European Genome-phenome Archive, trimmed using trimmomatic (default settings) and aligned to the hg19 reference genome using STAR aligner[9] with the –outFilterMultimapScoreRange 1 setting. Bam files were sorted, deduplicated as before and an mpileup file for the 19 preselected editing sites was generated using samtools. Varscan2 mpileup2snp was used for variant calling at these positions.

The “survminer”, which uses a maximally selected rank statistic to calculate the cutoffs and “survival” R packages were used for survival plots and uni/multivariate analysis, respectively.

Biological pathways were assigned to particular genes using genecards ([www.genecards.org](http://www.genecards.org) [10, 11]). Gene ontology (GO) enrichment analysis and KEGG pathway enrichment analysis were performed using the goana and kegga functions of edgeR (v3.26.8), respectively.

RNA editing was investigated by Sanger sequencing of selected target sites by PCR on first-strand cDNA (iScript, Biorad) using the primers listed in supporting table S2 and the BigDye Sequencing Kit (ThermoFisher).

Graphs were created in R and using Graphpad Prism 5. All figures were finalized using Inkscape0.48.

Western Blotting

Western blots were performed on cell lysates of isolated CLL cells (untouched MACS; Miltenyi Biotech) of untreated CLL patients and MEC1 cell lines with anti-ADAR1 Antibody (Picoband, PB9976, BOSTER) for detection of ADAR1 isoform p150 and p110 and anti-Pan-Actin (D18C11, #8456, Cell Signaling) as a control.

Statistical analysis

Statistical analyses were performed using Graph Pad Prism Version 5.02 (GraphPad Software, Inc.) or in R using statistical tests indicated in the respective figure legends. In case sample size was too small for calculating normal distribution, two-tailed unpaired t-tests were used. If samples were not normally distributed, two-tailed Mann-Whitney test or unpaired t test with Welch correction was used as indicated. Variances were calculated as indicated in figure legends. No statistical analyses for sample size estimates were used. No blinding or randomization was used. All available samples from the CLL cohorts were included in the study. Sample sizes, statistical testing, are indicated in each figure legend. N-values are independent experiments.

References

1. Stacchini, A., et al., *MEC1 and MEC2: two new cell lines derived from B-chronic lymphocytic leukaemia in prolymphocytoid transformation.* Leuk Res, 1999. 23(2): p. 127-36.

2. Langmead, B. and S.L. Salzberg, *Fast gapped-read alignment with Bowtie 2.* Nat Methods, 2012. 9(4): p. 357-9.

3. Bolger, A.M., M. Lohse, and B. Usadel, *Trimmomatic: a flexible trimmer for Illumina sequence data.* Bioinformatics, 2014. 30(15): p. 2114-20.

4. Kim, D., et al., *TopHat2: accurate alignment of transcriptomes in the presence of insertions, deletions and gene fusions.* Genome Biol, 2013. 14(4): p. R36.

5. Liao, Y., G.K. Smyth, and W. Shi, *featureCounts: an efficient general purpose program for assigning sequence reads to genomic features.* Bioinformatics, 2014. 30(7): p. 923-30.

6. Robinson, M.D., D.J. McCarthy, and G.K. Smyth, *edgeR: a Bioconductor package for differential expression analysis of digital gene expression data.* Bioinformatics, 2010. 26(1): p. 139-40.

7. Li, H., et al., *The Sequence Alignment/Map format and SAMtools.* Bioinformatics., 2009. 25(16): p. 2078-2079.

8. Koboldt, D.C., D.E. Larson, and R.K. Wilson, *Using VarScan 2 for Germline Variant Calling and Somatic Mutation Detection.* Curr.Protoc.Bioinformatics., 2013. 44: p. 15-17.

9. Dobin, A., et al., *STAR: ultrafast universal RNA-seq aligner.* Bioinformatics, 2013. 29(1): p. 15-21.

10. Rebhan, M., et al., *GeneCards: a novel functional genomics compendium with automated data mining and query reformulation support.* Bioinformatics, 1998. 14(8): p. 656-64.

11. Stelzer, G., et al., *The GeneCards Suite: From Gene Data Mining to Disease Genome Sequence Analyses.* Curr Protoc Bioinformatics, 2016. 54: p. 1 30 1-1 30 33.
