# Supplementary material for: RNA editing contributes to epitranscriptome diversity in chronic lymphocytic leukemia
Source: Leukemia. 2020 Jul 30;35(4):1053–63. doi: 10.1038/s41375-020-0995-6 (PMC8024191; doi:10.1038/s41375-020-0995-6)
Supplement: Supplementary file 2 — supplemental figure and table legends [file 41375_2020_995_MOESM2_ESM.docx]

Supplementary figures and tables

Supplementary figures S1-S9

Supporting Fig S1. Percentage of RNA-DNA single nucleotide differences (RDDs) in Alu and non-Alu sites and within non-synonymous editing sites in CLL cells from samples shown in Fig 1.

Supporting Fig S2. Correlation and linear regression analysis of editing frequency and expression levels of edited genes in the AGMT-REVLIRIT CLL cohort. R squares (goodness of fit) and p-values (significance that slope is not zero) are indicated within the graphs.

Supporting Fig S3. (A) Heat map of editing depths of 15 recurrent, recoding A>I editing sites in the CLL cohort and normal B cell subsets from Ferreira et al[19]. Shown are differences of editing depths between CLL and normal B cell subsets. Significances were determined by unpaired t-test. (B) Alu editing index (AEI) of CLL and normal B cell subsets and (C) of CLL cells according to IGHV mutation status. Significances were determined by unpaired t-test with Welch’s correction, assuming unequal variances. (D) Expression of ADAR isoforms p110 and p150 is shown for IGHV mutated and unmutated CLL samples and normal B cell subsets. P-values for IGHV mutated vs unmutated samples are indicated. p-values above normal B cell subsets indicate significance compared to CLL samples (irrespective of IGHV status). Significances were determined by unpaired t-test with Welch’s correction, assuming unequal variances.

Supporting Fig S4. Correlation and linear regression analysis of editing frequency and expression levels of edited genes in the Ferreira cohort. R squares (goodness of fit) and p-values (significance that slope is not zero) are indicated within the graphs.

Supporting Fig S5. Correlation and linear regression analysis of editing frequency and expression levels of edited genes in normal B cells. R squares (goodness of fit) and p-values (significance that slope is not zero) are indicated within the graphs.

Supporting Fig S6. (A) Alu editing index (AEI) of CLL samples assigned to editing clusters defined in Fig 3A, compared to normal B cell subsets. (B) Expression of ADAR isoforms p110 and p150 is shown for CLL samples within the respective clusters c1-c4. Significances determined by unpaired t-test with Welch’s correction, assuming unequal variances.

Supporting Fig S7. RNA editing in the AGMT-REVLIRIT CLL cohort. (A) Hierarchical clustering of recurrently edited sites from the CLL AGMT-REVLIRIT cohort[17] yields 7 editing clusters. (B) Left graph, time to first treatment of patients assigned to the 7 RNA editing clusters (color code according to A). On the right graph, non-cluster 5 patients were combined. Univariate analysis for cluster 5 vs non-cluster 5 patients is indicated in graph.

Supporting Fig S8. RNA editing in the AGMT-REVLIRIT CLL cohort according to IGHV mutation status. Time to first treatment from diagnosis (TTFT) was calculated for RNA editing cluster 5 patients versus non-cluster 5 patients in IGHV mutated and unmutated samples. Univariate analysis for cluster 5 vs non-cluster 5 patients is indicated in graph.

Supporting Fig S9. Progression free survival in CLL cohorts. (A) Progression free survival in CLL patients from the Ferreira cohort. Univariate analysis for cluster 1 vs non-cluster 1 patients is indicated in graph. (B) Progression free survival in CLL patients from the AGMT-REVLIRIT cohort. Univariate analysis for cluster 5 vs non-cluster 5 patients is indicated in graph

Supporting Fig S10. ADAR p110 and p150 isoform expression in MEC1 cells. (A) p110 and p150 levels in MEC1 cells calculated from RNAseq data. (B) Western Blotting of lysates from 8 randomly selected CLL samples and MEC1 and MEC1 ADAR knockout cells, using antibodies specific for ADAR and actin as a loading control.

Supplementary tables S1-S12

Supporting table S1. Patient details

Supporting table S2. Primer list

Supporting table S3. Expression of editing cofactors in the AGMT-REVLIRIT CLL cohort.

Supporting table S4. IGHV specific expression of recurrent editing sites within the AGMT-REVLIRIT CLL cohort.

Supporting table S5. Multivariate analysis within the Ferreira CLL cohort.

Supporting table S6. Multivariate analysis within the AGMT-REVLIRIT CLL cohort.

Supporting table S7. Expression of editing cofactors in the Ferreira CLL cohort.

Supporting table S8. Expression of RNA binding proteins in the Ferreira cohort.

Supporting table S9. Editing in MEC1 and MEC1 ADAR knockout cells of sites defined in glioblastoma cell line U87MG and breast cancer samples by Bahn et al, Genome Res. 2012 Jan;22(1):142-50.

Supporting table S10. Gene expression differences in MEC1 versus MEC1 ADAR knockout cells.

Supporting table S11. Different gene ontology pathways in MEC1 and MEC1 ADAR knockout cells.

Supporting table S12. Different KEGG pathways in MEC1 and MEC1 ADAR knockout cells.
