# Supplementary figures and images for: RNA editing contributes to epitranscriptome diversity in chronic lymphocytic leukemia
Source: Leukemia. 2020 Jul 30;35(4):1053–63. doi: 10.1038/s41375-020-0995-6 (PMC8024191; doi:10.1038/s41375-020-0995-6)

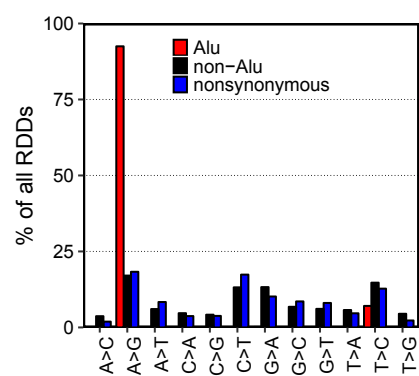

**Figure S1**

Supplement: Supplementary file 3 — Figure S1 [file 41375_2020_995_MOESM3_ESM.pdf]

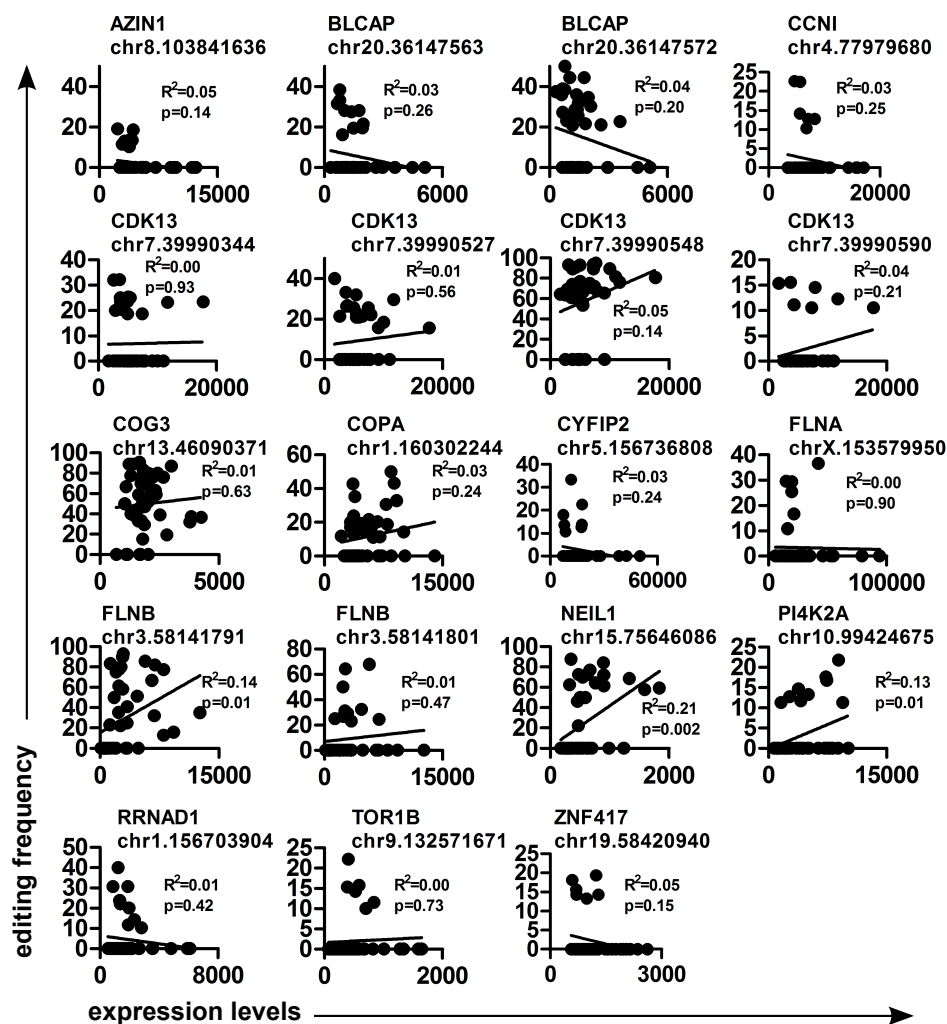

Figure S2

Supplement: Supplementary file 4 — Figure S2 [file 41375_2020_995_MOESM4_ESM.pdf]

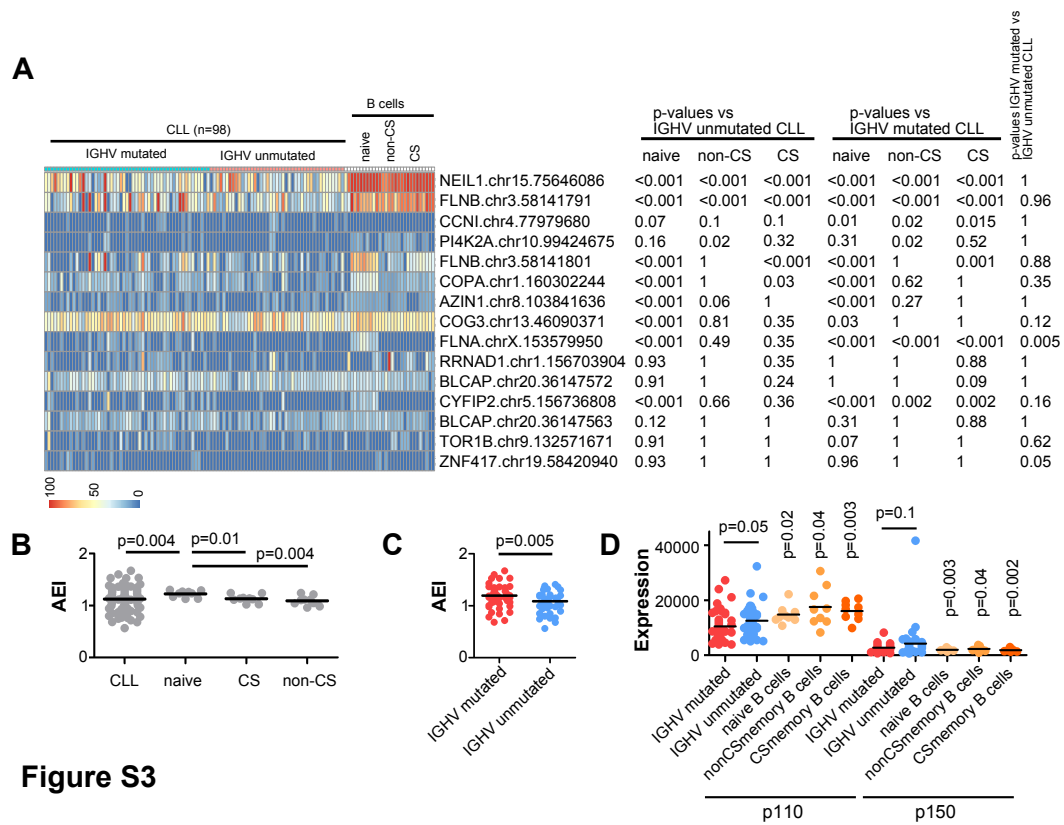

**Figure S3**

Supplement: Supplementary file 5 — Figure S3 [file 41375_2020_995_MOESM5_ESM.pdf]

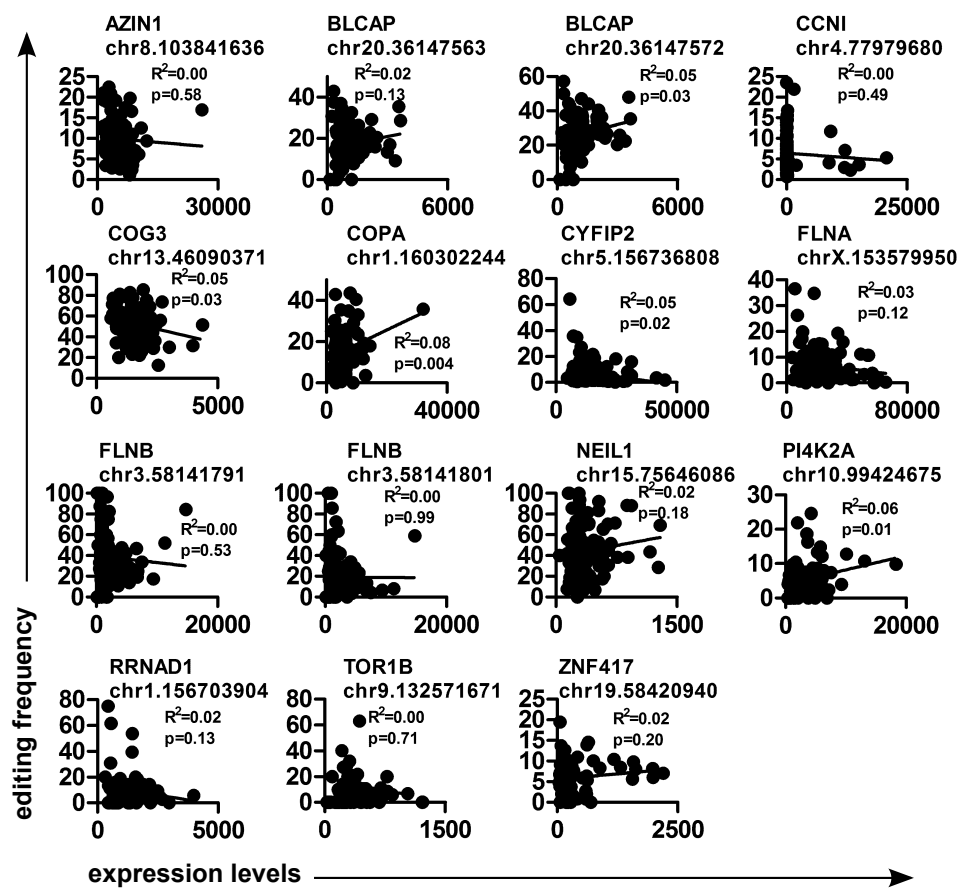

Figure S4

Supplement: Supplementary file 6 — Figure S4 [file 41375_2020_995_MOESM6_ESM.pdf]

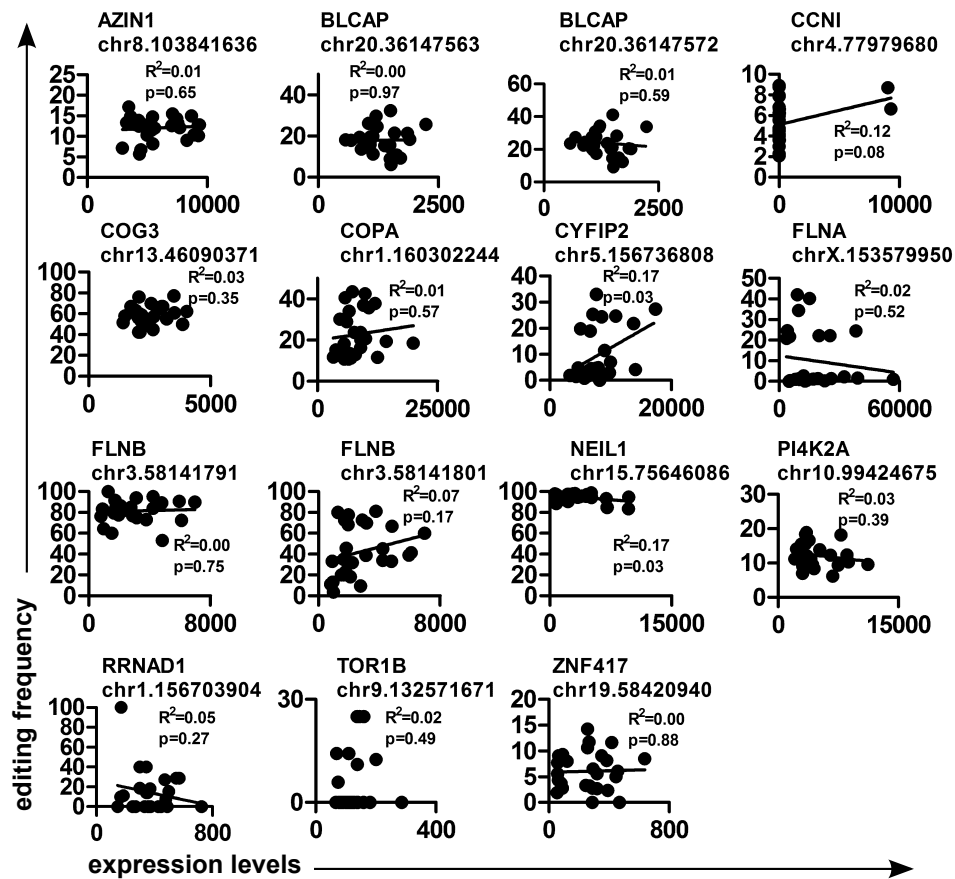

Figure S5

Supplement: Supplementary file 7 — Figure S5 [file 41375_2020_995_MOESM7_ESM.pdf]

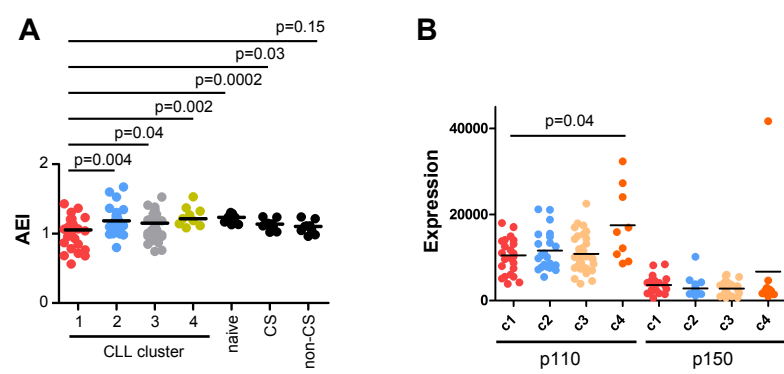

**Figure S6**

Supplement: Supplementary file 8 — Figure S6 [file 41375_2020_995_MOESM8_ESM.pdf]

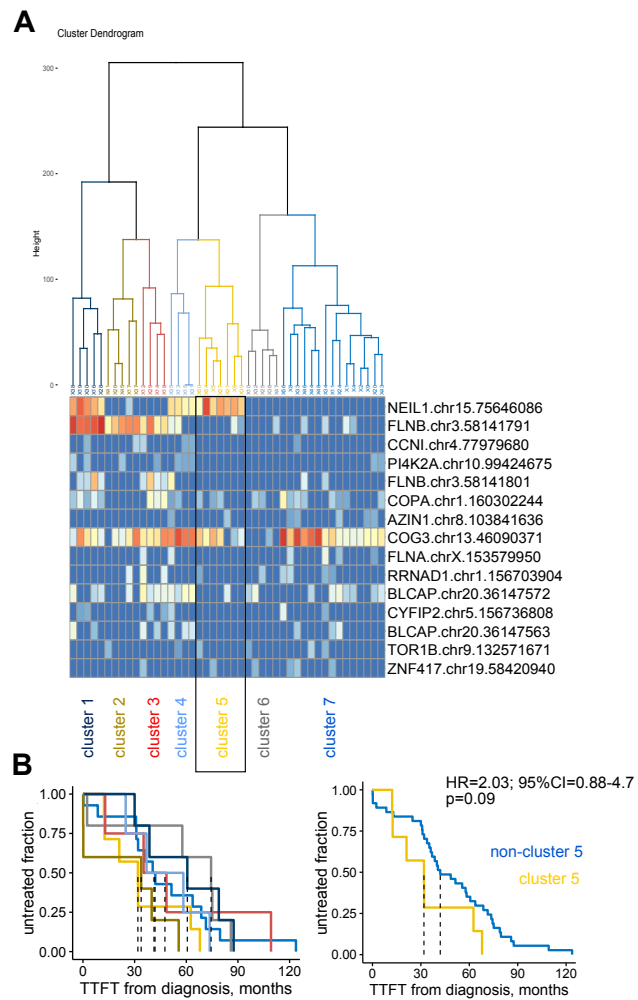

**Figure S7**

Supplement: Supplementary file 9 — Figure S7 [file 41375_2020_995_MOESM9_ESM.pdf]

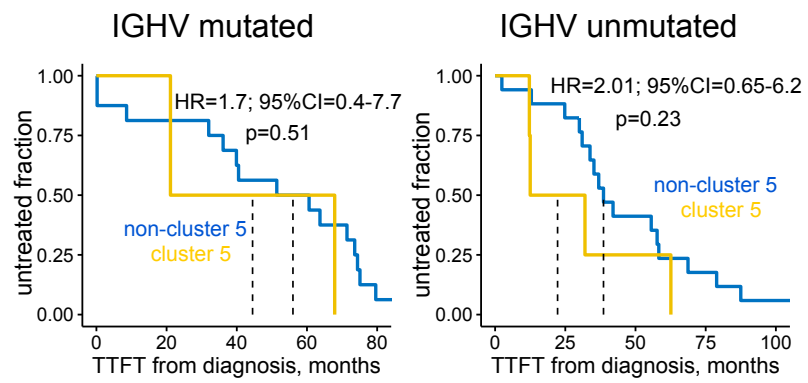

**Figure S8**

Supplement: Supplementary file 10 — Figure S8 [file 41375_2020_995_MOESM10_ESM.pdf]

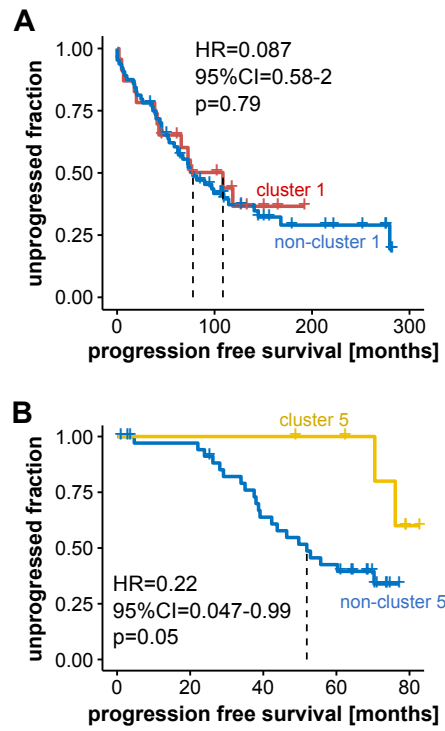

**Figure S9**

Supplement: Supplementary file 11 — Figure S9 [file 41375_2020_995_MOESM11_ESM.pdf]

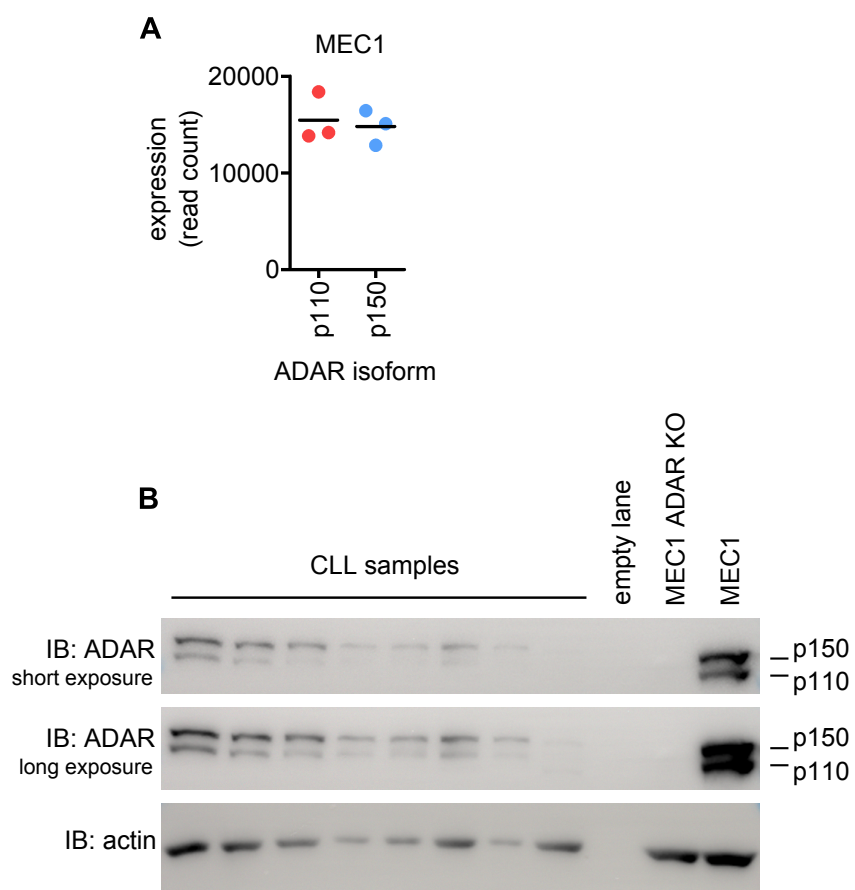

**Figure S10**

Supplement: Supplementary file 12 — Figure S10 [file 41375_2020_995_MOESM12_ESM.pdf]
