# Supplementary material for: RNA editing contributes to epitranscriptome diversity in chronic lymphocytic leukemia
Source: Leukemia. 2020 Jul 30;35(4):1053–63. doi: 10.1038/s41375-020-0995-6 (PMC8024191; doi:10.1038/s41375-020-0995-6)
Supplement: Supplementary file 16 — Table S4 [file 41375_2020_995_MOESM16_ESM.docx]

| RNA editing site | p-value IGHV mutated vs unmutated |
| --- | --- |
| FLNB;chr3:58141791 | 0.237 |
| FLNB;chr3:58141801 | 0.544 |
| NEIL1;chr15:75646086 | 0.819 |
| AZIN1;chr8:103841636 | 1 |
| BLCAP;chr20:36147563 | 1 |
| BLCAP;chr20:36147572 | 1 |
| CCNI;chr4:77979680 | 1 |
| CDK13;chr7:39990344 | 1 |
| CDK13;chr7:39990527 | 1 |
| CDK13;chr7:39990548 | 1 |
| CDK13;chr7:39990590 | 1 |
| COG3;chr13:46090371 | 1 |
| COPA;chr1:160302244 | 1 |
| CYFIP2;chr5:156736808 | 1 |
| FLNA;chrX:153579950 | 1 |
| PI4K2A;chr10:99424675 | 1 |
| RRNAD1;chr1:156703904 | 1 |
| TOR1B;chr9:132571671 | 1 |
| ZNF417;chr19:58420940 | 1 |

Supporting table S4
