# Supplementary material for: RNA editing contributes to epitranscriptome diversity in chronic lymphocytic leukemia
Source: Leukemia. 2020 Jul 30;35(4):1053–63. doi: 10.1038/s41375-020-0995-6 (PMC8024191; doi:10.1038/s41375-020-0995-6)
Supplement: Supplementary file 17 — Table S5 [file 41375_2020_995_MOESM17_ESM.docx]

Ferreira cohort (Ref 20), multivariate analysis

Parameter HR (95%CI) p-value

Editing cluster 1 1.1 (0.62 − 2.1) 0.663

tri12 7 (0.80 − 3.6) 0.169

del17p 2.7 (0.78 − 9.2) 0.116

del13q 1.5 (0.80 − 2.8) 0.203

del11q 2.1 (0.94 − 4.8) 0.07

IGHV_status 3.3 (1.81 − 5.9) <0.001

Editing cluster 1: high Neil1 editing, intermediate COG3 editing, low editing of other sites

Supporting table S5
