# Supplementary material for: RNA editing contributes to epitranscriptome diversity in chronic lymphocytic leukemia
Source: Leukemia. 2020 Jul 30;35(4):1053–63. doi: 10.1038/s41375-020-0995-6 (PMC8024191; doi:10.1038/s41375-020-0995-6)
Supplement: Supplementary file 18 — Table S6 [file 41375_2020_995_MOESM18_ESM.docx]

Revlirit cohort (Ref 18), multivariate analysis

Parameter HR (95%CI) p-value

Editing cluster 5 1.9 (0.77 − 4.9) 0.158

del17p or del11q 1.1 (0.44 − 2.6) 0.864

IGHV_status 1.2 (0.54 – 2.5) 0.687

Editing cluster 5: high Neil1 editing, intermediate COG3 editing, low editing of other sites

Supporting table S6
